# Adenylyl Cyclase 9: A Maverick Signalling Act in Search of Plots

**DOI:** 10.3390/cells15201836

**Published:** 2026-10-09

**Authors:** Ferenc A. Antoni

**Affiliations:** Centre for Discovery Brain Sciences, Institute for Neuroscience and Cardiovascular Research, University of Edinburgh, Edinburgh EH16 4TJ, Scotland, UK; ferenc.antoni@ed.ac.uk; Tel.: +447968647338

**Keywords:** protein phosphorylation, calcineurin, adenylyl cyclase, cyclic AMP, protein kinase, AKAP, Rap1, G proteins

## Abstract

Cryo-electronmicroscopy has revolutionized the study of integral membrane proteins. In the case of adenylyl cyclase 9 (AC9)—one of nine transmembrane paralogues in mammals that produce the ubiquitous intracellular messenger cyclic AMP—the technique was instrumental in revealing the structural basis of a biological paradox, namely, that AC9 is strongly autoinhibited when in a complex with its physiological stimulus, receptor-activated Gsα. Furthermore, AC9 is also insensitive to inhibitory G proteins because it lacks the requisite binding pocket. As AC9 is widely distributed in the body, this regulatory conundrum has potentially broad implications. However, deletion of the *adcy9* gene in mice causes a high level of pre-weaning lethality. Hence, our knowledge regarding the biological role of AC9 in mammals is rudimentary at best. In mammalian AC9, autoinhibition is modulated by post-translational modifications, including protein phosphorylation. However, AC9 has over 70 documented phosphorylations, the majority of them on serines in isoform-specific disordered domains. What is their functional relevance—if any? This paper reviews the current knowledge regarding the regulation of AC9, a maverick amongst transmembrane adenylyl cyclases.

## 1. Introduction

Since the discovery of the second messenger concept and adenosine 3′:5′ monophosphate (cAMP) as a newly synthesized mediator [1], there have been innumerable studies analyzing the production, metabolism and actions of cAMP. The core finding that cAMP is primarily a mediator of extracellular signals is deeply entrenched in textbooks. However, the advent of molecular cloning and the discovery of the genetic diversity of proteins involved in cAMP signalling [2,3] has started to change this thesis. This paper will focus on the production of cAMP and will highlight some of the regulatory features of adenylyl cyclase 9 (AC9) that point us away from the conventional receptor-G protein-adenylyl cyclase pathway. It will serve to underpin the change in nomenclature from “second messenger” to “intracellular signalling molecule”. Admittedly even this designation has exceptions that strengthen the rule as cAMP is an intra- as well as extracellular messenger in the social amoeba *Dyctiostelium* [4].

### 1.1. Some of the Discoveries Relevant to AC9

**Figure 1:**
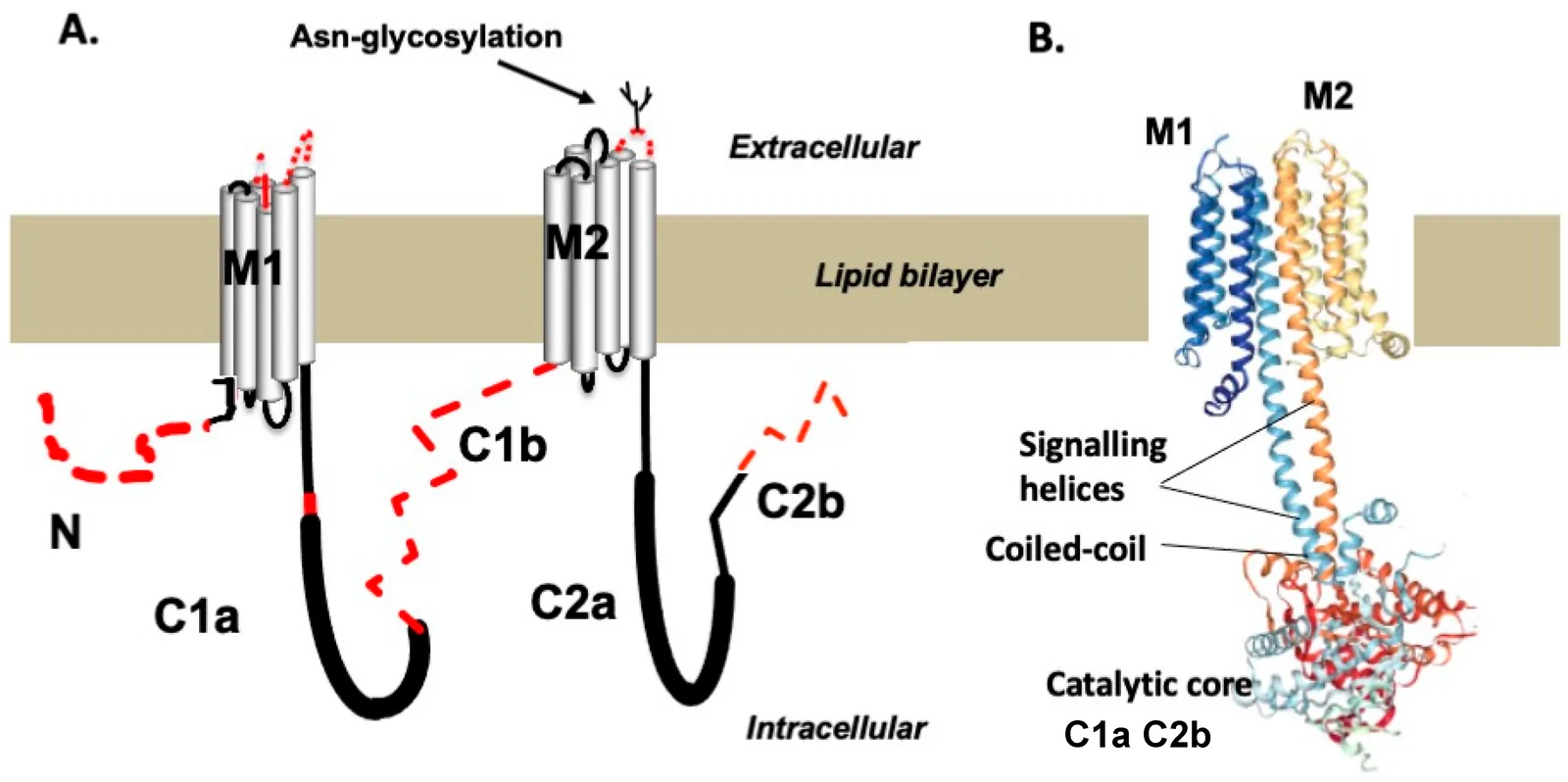
(**A**) Schematic representation of the single polypeptide of AC9; the dashed red lines indicate lack of representation in the cryo-EM images of the protein. The thicker lines show the C1a and C2a catalytic domains; these show significant sequence homology with other transmembrane AC9 paralogues. N—N terminal domain, C1b and C2b—cytoplasmic loops, M1 and M2—transmembrane arrays. These all have isoform-specific primary sequences. (**B**) The cartoon representation of the tripartite structure of AC9 produced by cryo-electronmicroscopy. Note presence of a parallel coiled-coil formed by the signalling helices above the catalytic core.

**Figure 2:**
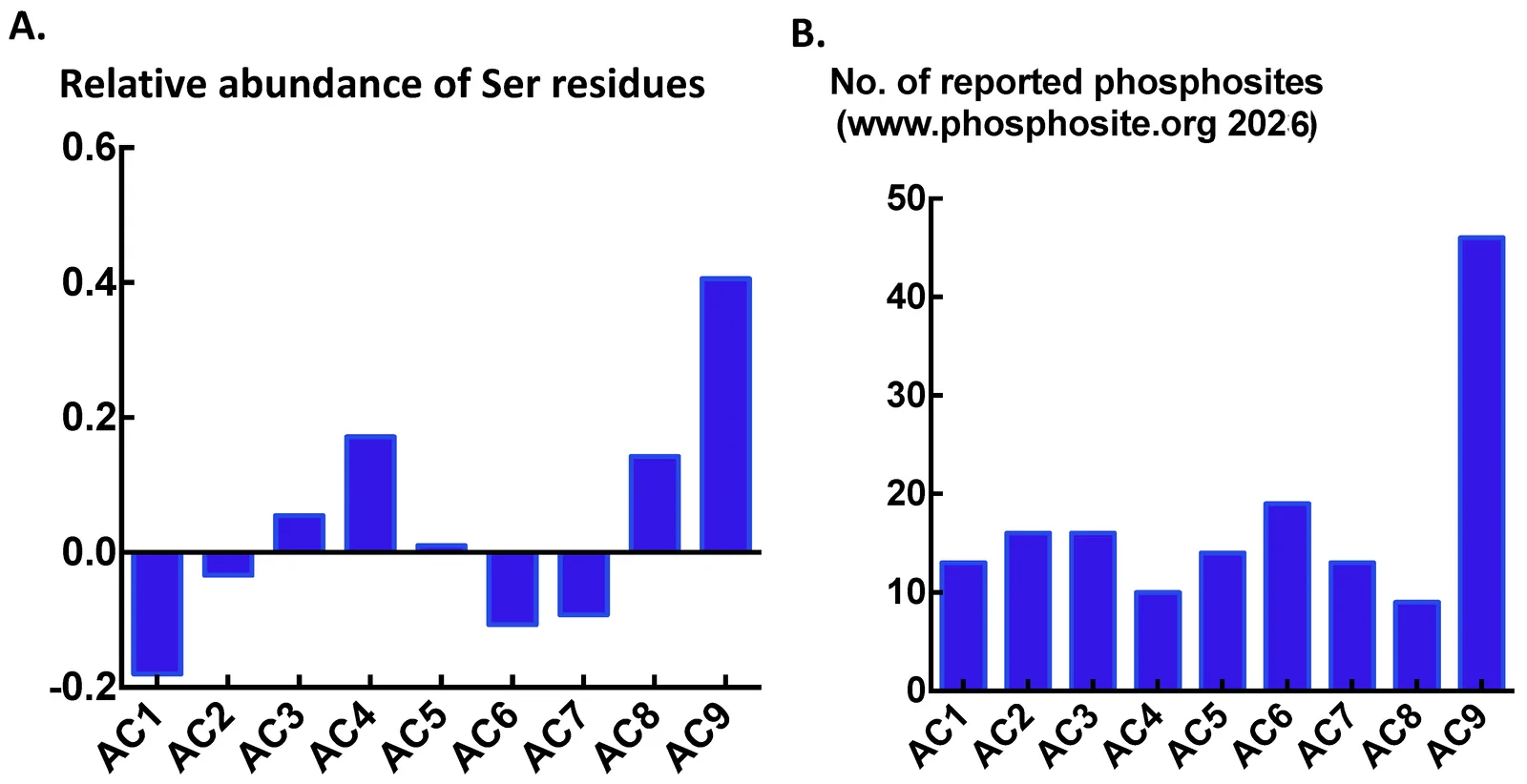
(**A**) When compared with other proteins, including other human transmembrane ACs, human AC9 has a conspicuously high percentage of serine residues. Formula of relative abundance: the percentage of serine residues relative to the average abundance (currently 7.30%) in proteins of the SwissProt database; 0 = average, + above average, − below average. (**B**) Accordingly, AC9 has by far a larger number of phosphorylations than the other mammalian transmembrane ACs. Total number of phosphorylations for human, mouse and rat ACs (source: https://www.phosphosite.org/, (accessed 25 July 2026)).

Adenylyl cyclase 9 is one of nine transmembrane ACs in the mammalian genome [2,3] (Figure 1). It was first discovered on the basis of pharmacologic analysis pointing to inhibition by the Ca^2+^/calmodulin activated protein phosphatase calcineurin (PPP3) [5,6]. A phosphoproteomic follow-up revealed at least 12 sites of phosphorylation of human AC9 expressed in HEK293 cells, making the identification of the relevant calcineurin sensitive sites difficult, especially in the absence of detailed structural information about the domains the phosphorylations were in [7]. However, it is clear from the protein statistical data at https://www.uniprot.org/uniprotkb/statistics (accessed 25 July 2026) that AC9 has a high abundance of serine residues when compared with its human paralogues (Figure 2A). Accordingly, https://www.phosphosite.org/lists (accessed 25 July 2026) over 40 phosphorylations of AC9 (Figure 2B). Notably, sites such as https://epsd.biocuckoo.cn/index.php (accessed 25 July 2026) list over 70 sites, the vast majority of which are phosphoserine. In addition to cloning and expression of the AC9 cDNA, subsequent breakthroughs were the discovery of bidirectional autoregulation [8,9] and the cryo-EM structures that demonstrated the tripartite molecular architecture and revealed the structural correlates of autoregulation [10,11]. Finally, it was recently found that cAMP may enhance the activity of AC9 through GTP exchange proteins activated by cAMP acting on Rap1 [12].

### 1.2. Biological Role of AC9

Deletion of the *adcy9* gene has been reported by two different methods. First, Lexington Genetics used a gene-trap approach (https://www.mmrrc.org/catalog/sds.php?mmrrc_id=11682 (accessed 25 July 2026)), and second, the Sanger Institute reported homology-arm based deletion using C57BL/6 stem cells [13]. Yet others have bred the gene-trap mutation onto the C57BL/6 background [14,15]. The outcome reported by all experimenters was that homozygous deletion of *adcy9* caused severe pre-weaning lethality, up to 90%. The reasons for this rate of post-natal attrition have not been investigated in detail, the Sanger group cited possible placental insufficiency [13]. In this context, it is worth mentioning that *adcy9* is a core gene of placental trophoblasts in mice [16] and the formation of syncytiotrophoblast in the placenta is highly dependent on cAMP signalling [17]. Given the high rate of early postnatal lethality of *adcy9(−/−)*, the adult survivors most likely reflect the probability of mobilizing compensatory changes that allow survival. Experimental approaches to testing adult *adcy9(−/−)* mice have been summarized previously [18].

As an alternative to mice, deletion of the *adcy9a* gene in zebrafish caused early heart failure in larvae 3 days after fertilization [19]. Whether or not this has any relevance for explaining the causes of preweaning lethality in mice has not been investigated. More recently, Wang et al. working with the palanarian *Dugesia japonica* reported that *adcy9* was essential for the regeneration of the cephalic ganglia [20]. The exact underlying mechanism was not established but circumstantial evidence was provided that *adcy9* down-regulates the expression of the *mitofusin-1* gene that induces the fusion of mitochondria [20]. It is of note that the planarian AC9 does not have a sizeable C2b domain and is unlikely to feature the autoregulation seen in vertebrate AC9 paralogues.

### 1.3. Evolution of the adcy9 Gene—Further Clues to Biological Functions

An ancestral AC9 is already evident in sponges and *C. elegans*. The *C. elegans acy-1* gene encodes an AC that has significant homology in the isoform specific regions with mammalian AC9. Importantly, the acy-1 protein has no C2b domain to speak of (Figure 1A), and it is also highly responsive to the diterpene drug forskolin. In contrast, vertebrate AC9s have a long C2b domain, comprising at minimum 111 amino acid residues, and a very low response to forskolin. Of further interest is that teleost-specific whole genome duplication resulted in two subfunctionalized AC9 genes (ohnologs) that have persisted throughout teleost evolution [21]. By comparison, 85–90% of the ohnologs arising upon genome duplication revert to a single gene [22]. The main difference between the teleost *adcy9* ohnologs is that *adcy9b* encodes an autoinhibited form of AC9 similar to the mammalian version. The *adcy9a* gene encodes an enzyme with a long C2b domain which lacks the autoregulatory motif and shows no autoinhibition of Gs-coupled receptor stimulation. Of further significance is the observation that the expression of *adcy9b* is largely restricted to the brain and the testis while *adcy9a* is expressed at substantial levels in all organs examined [21]. It is thus conceivable that autoinhibition is an evolutionary burden in some tissues, above all the mammalian myocardium, where it seems to be eliminated by proteolytic cleavage of AC9 [23].

### 1.4. Tissue Distribution, Regulation of Expression and Subcellular Targeting

AC9 mRNA is widely expressed in the body [3,18]. The brain, heart, skeletal muscle, endocrine organs, the aorta and the prostate all express high levels of AC9 mRNA, whereas the spleen and thymus have low levels. Within the brain, all types of cells appear to express AC9, with particularly high levels of protein in hippocampal neurons and layers II and III in the cingulate cortex [18,24]. Mouse spinal cord motoneurons also express high levels of AC9 mRNA (https://mousespinal.brain-map.org/ (accessed 25 July 2026)). Of further interest is that mapping of human glioma mRNA expression shows high levels of AC9 mRNA in the leading edges of the tumours as well as in the infiltrating vasculature (https://glioblastoma.alleninstitute.org (accessed 25 July 2026)).

The 5′ and 3′ untranslated regions (UTR) of the *ADCY9* gene have not been analyzed in detail. In human breast cancer cells, ADCY9 is a bona fide estrogen-induced gene [25]. A study examining the genes regulated by mouse *achaete–scute homologue 1 (ascl1)* reported that this transcription factor is an important regulator of *ADCY9* expression in neuroendocrine cells of the prostate gland [26]. The expression of AC9 in neural stem cells [20] is in line with the control of its expression by *ascl1*. Regarding the 3′ UTR, micro RNAs 142 and 181 are established regulators of *ADCY9* expression, and the suppression of AC9 translation by miRNA142-3p has been implicated, amongst others, in immune and cardiovascular regulation [18].

It is assumed that ACs are largely localized to the plasma membrane, where they are concentrated and ready for interaction with Gs-alpha. This concept is supported in HEK293 cells and other epithelial cell lines, where transfected ACs, including AC9, appear predominantly in the plasma membrane [27]. However, the situation may be different in neurons where the bulk of adenylyl cyclase appears to localize to discrete punctae in the cytoplasm [27]. Within nerve cells of the adult rodent brain, AC9 appears to be restricted to the somatodendritic compartment and is also found throughout the cytoplasm with no obvious enrichment in the plasma membrane [18]. In this context it is worth recalling the structural features of mammalian adenylyl cyclases: the cytoplasmic domains are sufficient for Gsα stimulated cAMP biosynthesis [3,18]. Thus, the cytoplasmic domain of a fully synthesized and folded adenylyl cyclase located in intracellular membrane compartments is, at least in principle, fully responsive to physiological stimulation provided Mg^2+^ and ATP are present in sufficient amounts. Indeed, it has been shown that upon receptor activation at the cell surface, Gsα translocates into the cell interior to home in on various targets [28]. More recently, it was reported in cultured striatal spiny neurons that when in complex with activated Gsα, AC9 is selectively endocytosed into early endosomes and continues to generate cAMP in the endoplasmic reticulum [29]. Moreover, these workers also reported differential localization of AC paralogs in the cilia of the neurons—AC9 was found outside of these protrusions.

### 1.5. Summary

Overall, the understanding of the biological role of AC9 is fragmented and incomplete. The preweaning lethality in mice presents an impasse that has not been overcome by tissue or cell-type selective gene deletion. Work in other species has provided intriguing and potentially important results, but it is unclear to what extent these are relevant to mammals. Thus, it is the unique regulatory features, tissue and subcellular distributions of AC9 that collectively point to a significant involvement in the maintenance of mammalian body homeostasis/allostasis.

## 2. Structure, Activity and Unique Features of Regulation

Some considerations on how to estimate cAMP production by AC9 are in order. Most groups use HEK293 or COS-7 cells transiently or stably transfected with AC9 [9,30,31,32,33]. Intact cells or crude membranes are analyzed for cAMP in endpoint assays such as radioimmunoassay, ELISA or the Cisbio time-resolved fluorescence assay. The respective differences between the AC9-expressing cells and skeleton-vector-expressing cells are taken as the cAMP attributable to AC9. Yet other labs have deployed FRET and BRET genetic sensors in intact cells—these probes offer the advantage of being ratiometric and supporting time-course experiments. In addition, some groups have knocked down endogenous AC expression in HEK293 cells [34] or resurrected HC-1 cells that have little or no transmembrane AC activity [12] to selectively monitor cAMP production by AC9. Given what is known about intracellular cAMP compartments (or nano-domains) [35], one would ideally be able to focus on selected intracellular domains and analyze the effects of mutations in situ [29]. However, as yet, this kind of methodology is not ready for the screening of larger pools of mutations.

In the following section, recent results from my laboratory are presented of intact cells with Glosensor 22F [36] developed at Promega as the cAMP reporter system. A stand-out dynamic range of up to 1000-fold was observed, especially in HEK293FT cells where the large T antigen drives the expression of the biosensor to levels where signal saturation is not a problem. This is in contrast to the BRET- and FRET-based probes, where the monitoring of basal as well as receptor-evoked cAMP levels in a single experiment proved to be a problem due to the rapid saturation of the signal. The main drawback of Glosensor 22F is that it relies on the Mg^2+^ATP of the host cells. Recent data indicate that intracellular ATP levels can fluctuate significantly [23]. Hence, metabolic impairment needs to be avoided. A further issue is standardization between groups of cells expressing different mutants of AC9.

While AC9 protein expression levels measured by immunoblots usually show low variability between mutants [8,9], the standardization for Glosensor22F expression levels is also important. In the case of AC9, one can exploit the low sensitivity of the enzyme to forskolin and the synergy of forskolin with Gsα on the host cell cyclases—usually AC6 [37,38]. Experiments with AC9-expressing cells can be standardized by applying a low concentration of forskolin (5 µM) and a broad-spectrum blocker of cyclic nucleotide phosphosphodiesterases such as iso-butyl-methyl-xanthine (IBMX) at the end of the experiment. While this can flatten agonist dose–response curves due to the non-linearity of the sensor at high cAMP concentrations [36], it provides a satisfactory measure of the cAMP-reporting capacity of each well in a plate-reader based assay. When judiciously applied, this method has worked well in HEK293FT, MCF-7 and AtT20 cells.

### 2.1. Autoregulation

**Figure 3:**
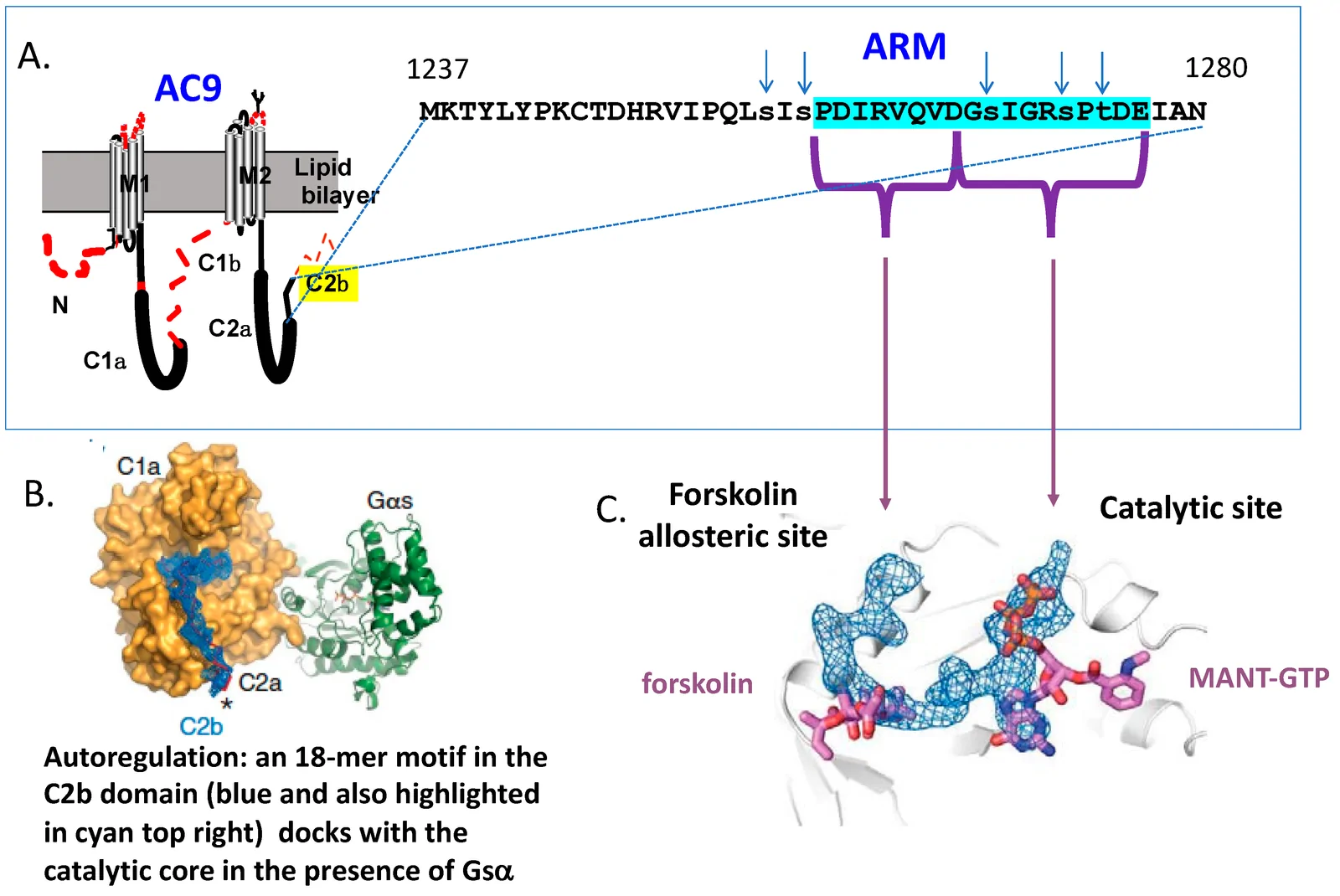
Schematic representations of the autoregulatory motif (ARM) of AC9. (**A**) The linear representation of AC9, indicating the position of the ARM and to the right the primary sequence of ARM (in cyan) and its environment. Note that AC9 at positions 1237–1280 is close to 100 percent conserved in the vertebrate subphylum [21]. The small arrows indicate phosphorylations reported in various databases. MANT-GTP: 2′/3′-O-(N-Methylanthraniloyl)-guanosine-5′-triphosphate used to stabilize the conformation of the active site of AC9 for cryo-electronmicroscopic analysis. (**B**) The surface of the catalytic core made up of the C1 and C2 domains in complex with Gsα. Note that in the presence of Gsα, the ARM depicted in blue is binding to the forskolin as well as the substrate binding site and exposed at the surface of the protein. (**C**) A schematic close-up of ARM as a wire outline diagram docked in the forskolin allosteric site and the ATP substrate site. Both forskolin and ATP (here MANT-GTP) are displaced by the ARM. The steric images were downloaded from Qi et al. [11].

**Figure 4:**
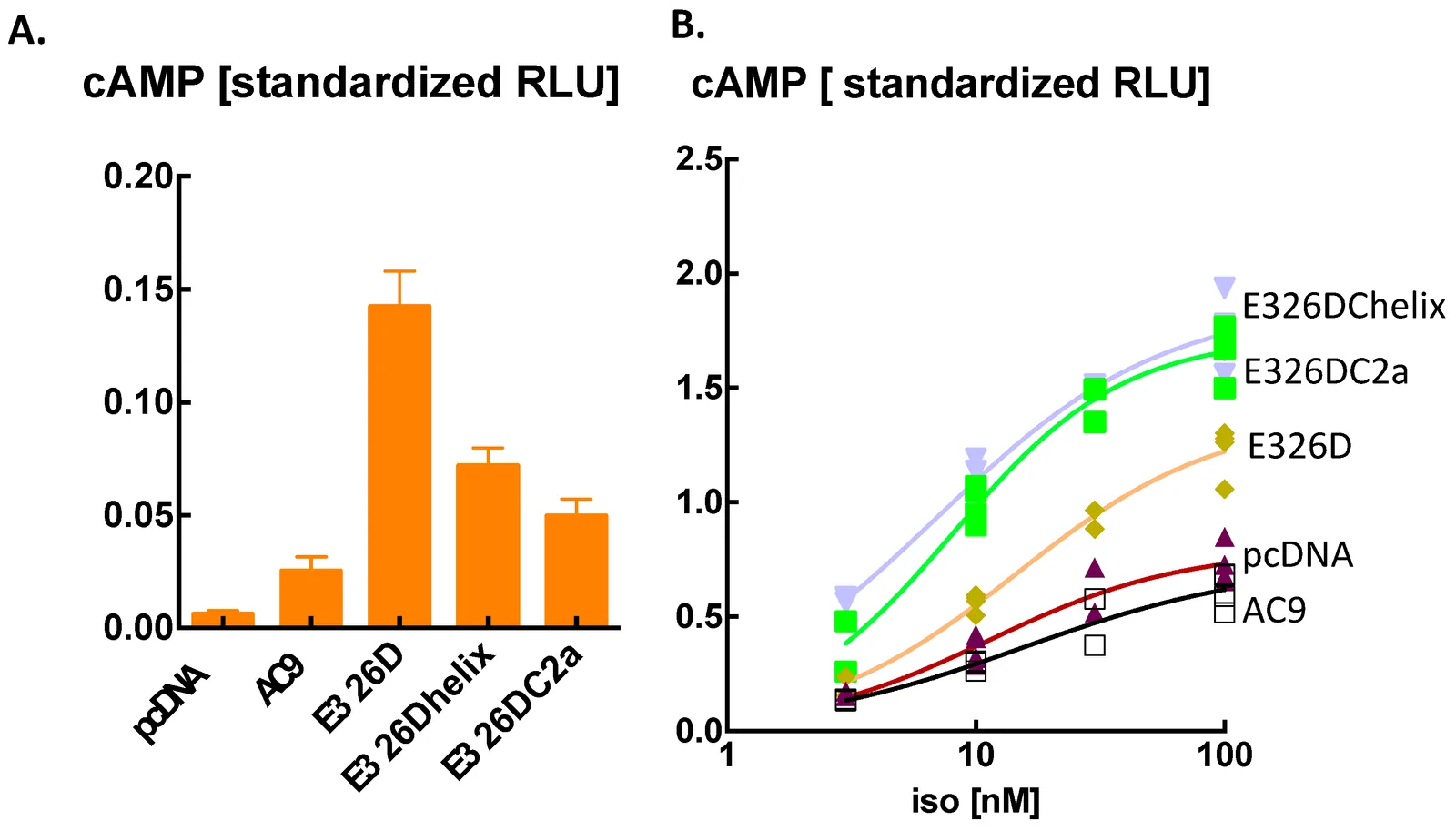
Analysis of the E326D mutation in the coiled-coil of AC9. E326Dhelix: In addition to E326D, the AC9 specific residues in 4αhelix of the C1a domain were changed to those of AC5, i.e., N515T, L519H, Q522A, L523G. E326DC2a has the C2b domain removed. cAMP levels were detected by Glosensor22F luminescence. Raw data were standardized by dividing with the RLU counts elicited by 5 µM forskolin/0.5 mM iso-butyl-methyl-xanthine-IBMX applied to each well. See ref. [9]. for further details. (**A**) Basal levels of cAMP. One-way ANOVA of log-transformed values F (4, 45) = 412.3, *p* < 0.0001 post hoc comparisons by Tukey’s test for multiple comparisons: the helix mutation reduced the effect of E326D (*p* < 0.001) which was further diminished by removing the C2a domain (*p* < 0.001). Notably, E326DC2a was significantly greater than AC9, *p* < 0.001). Means ± S.D. (**B**) Effect of isoproterenol (iso). In contrast to the effects on basal levels, E326Dhelix and E326DC2a produced significantly more cAMP at 10 and 100 nM iso than E326D. In turn, E326D-expressing cells produced significantly more cAMP than pcDNA- or AC9-expressing ones. One-way ANOVAs, followed by Tukey’s test for multiple comparisons: n = 4/group. All data points are shown, unpublished results.

The stand-out regulatory feature of vertebrate type AC9 is autoregulation. First, it was shown that a short, linear autoregulatory motif (ARM) in the C2b domain markedly inhibited the activation of AC9 by Gs-coupled receptors [9]. The same motif was shown to occupy the substrate binding site of the enzyme in the presence of activated Gsα and also to dock to the forskolin-sensitive positive allosteric site (Figure 3). [10,11]. The N-terminal half of ARM interacts with the forskolin site and the C-terminal end with the substrate binding site (Figure 3). The interaction with the forskolin site raised the possibility that the C2b domain could also stimulate the activity of AC9. This was experimentally verified by examining the effects of a point mutation—E326D—in the coiled-coil that is formed by the signalling helices above the catalytic core [8]. This mutation can be expected to destabilize the coiled coil [39]. Further experiments confirmed that E326D has a marked stimulatory effect on basal cAMP production and also enables the response to Gs-coupled receptor stimulation (Figure 4). The enhancement of basal cAMP production by E326D was attenuated by converting the AC9 forskolin binding-pocket at the 4-αhelix of the C1a domain into that of AC5 (Figure 4A). It was reduced further, but still significantly above the level of wild type AC9, upon deletion of the entire C2b domain (Figure 4A). The results confirm previous work [8] showing that the coiled-coil has a tonic inhibitory effect on catalytic activity. This is reduced upon destabilization of the coiled-coil, resulting in an increase in the intrinsic activity of the enzyme, which is amplified further by the N-terminal part of the ARM that interacts with the forskolin-positive allosteric site. Thus, the ARM has positive allosteric (forskolin-like) activity, as anticipated on the basis of the cryo-EM structure.

Analysis of the E326D effect on the response to isoproterenol confirmed that it enabled stimulation by Gsα and that the ensuing enhancement of cAMP biosynthesis is still modulated by autoinhibition from ARM (Figure 4B). The surprising finding here was that the 4-αhelix interaction with ARM was required for effective autoinhibition of Gsα-induced cAMP production, whereas it markedly enhanced the effect of coiled-coil destabilization on basal activity. However, the recent results by the Korkhov group support precisely this finding [40]. They contend that interaction of ARM residues R1263 and V1266 with the 4-αhelix at residues Q522 and N515, respectively, blocks the access of ATP to the substrate binding site. Notably, residues Q522 and N515 of the 4-alpha helix are unique to AC9. This result lends functional support to the observation that the enzyme conformations at 4-αhelix during basal and Gsα stimulated activity are markedly different [10,40].

With respect to autoinhibition, the essence of its mechanism is the insertion of the C-terminal half of ARM into the active site of the enzyme [10,11]. The results of Qi et al. [11] indicated that it is a competitive inhibition of ATP access resulting in a 40-fold increase in the Km. This leads to some important considerations regarding the effective concentrations of ATP available to AC9. The Km of AC9 for ATP is around 300 µM, while the intracellular concentration of ATP is thought to be around 2 mM or greater [23,41,42]. The dynamics and compartmentation of intracellular ATP levels is a burgeoning field beyond the scope of this review [43]. Moreover, as the AC activity was monitored in the presence of Mn^2+^, which also seems to change the Km for ATP [44], the results may have different quantitative outcomes without this ion being present. Suffice it to say that during cellular activity, intracellular ATP levels vary significantly and could prove rate-limiting to autoinhibited AC9 [42,45]. This topic is clearly ripe for further investigation.

Regardless of the exact mechanism of autoinhibition, it is also apparent that there are processes by which it is reduced. First, in rodents and human samples, a major shortened AC9 species is found in cardiac membranes that have no C2b immunoreactivity [9,14]. In the absence of evidence for mRNA splicing and editing events, the parsimonious explanation for the C-terminally truncated AC9 is proteolytic cleavage of AC9 in the heart [9]. Furthermore, it can be argued that autoinhibition of AC9 is an evolutionary burden for cardiovascular function [23,46].

A plausible second type of mechanism modulating autoinhibition is protein phosphorylation. As highlighted in Figure 3, phosphoserines at positions 1257, 1259, 1269, and 1273 and phosphothreonine at 1275 have been reported (https://phosphosite.org (accessed 25 July 2026)) [47]. Examination of the potential steric interactions of S1269, S1273 and S1275 in the currently available cryo-EM poses suggests that the introduction of large negative charges to these residues will lead to a disruption of the interaction between ARM and the C2a domain. The same poses also indicate that these residues are exposed at the surface of the protein (Figure 3), and thus are potential substrates for protein kinases. Once more, this is a topic suitable for future work.

### 2.2. Phosphorylation

As already outlined in the preceding sections, AC9 contains a large percentage of serine residues and a large number of registered phosphorylations in databases. The most comprehensive of these is https://epsd.biocuckoo.cn/index.php (accessed 25 July 2026). Of further note is a recent paper charting the protein phosphorylations in about 1000 samples of solid cancers [47]. This found 22 Ser/Thr phosphorylations in human AC9, and, most remarkably, no phosphorylated tyrosines [47]. Overall, the phosphorylations in AC9 can be grouped into six clusters: N-terminal domain, the TM6 signalling helix below the coiled coil, two clusters in the C1b domain, the ARM and the remaining segment of the C2b domain. Serine being a significant disorder inducing amino acid in proteins, it is not altogether surprising that the vast majority of these phosphorylations are found in the disordered domains of AC9 that are isoform specific. As the disordered domains are very sparingly represented in the cryo-EM maps, precious little information can be gleaned from the available structural studies as per the potential impact of these phosphorylations. The best structural information is available for the ARM, as its interaction with the C1a and C2a domains in the presence of Gsα has been captured by both groups reporting the cryo-EM structure, as has been discussed in the preceding sections (also, see Figure 3). Finally, it is of note that a carefully conducted study in differentiated HL-60 cells provided evidence that AC9 is stimulated by a pathway involving the activation of PKC-beta by mTORC2 and is an important mediator of chemotactic mobility [48]. However, the sites of phosphorylation on AC9 were not identified in this study.

### 2.3. Protein–Protein Interactions

#### 2.3.1. Stimulatory G Protein (Gs)

The classic physiologic stimulus for ACs is activation of cell surface receptors coupled to Gs [49]. The activated GTP-Gsα complex derived from Gs clearly stimulates AC9 in crude membrane preparations, while other G protein subunits have no significant effect [11,31,50]. The full-length bovine AC9 could only be stabilized for cryo-EM imaging in complex with Gsα or an artificially produced protein that had Gsα-like effects [11,51]. The structural details of the Gsα–AC9 interaction have been mapped for bovine as well as human AC9 [10,51]. As to why the autoinhibition of Gsα evoked cAMP production by AC9 has been only demonstrated in intact cells or membranes assayed in the presence of Mn^2+^, this remains to be clarified.

#### 2.3.2. Ras-Related Protein 1 (Rap1)

A recent study reported that the small GTPase Rap1 can stimulate human AC9 in a manner dependent on cyclase-associated protein 1 (CAP1) [12]. Intriguingly, the CAP1 effect appeared to be specific for AC9. Furthermore, it was shown that Rap1 physically interacts with the C2a catalytic domain of AC9. Of further interest is the finding that the interaction with Rap1/GTP_CAP1 enhanced the efficacy of forskolin to activate AC9 [12]. Given the results with E326D shown in Figure 4, it is tempting to speculate that the physiologic ligand potentiated by Rap1GTP_CAP1 is the N-terminal part of ARM that has forskolin-like activity. Of further note is that the GTPase activity of Rap1 is stimulated by EPAC2 and NCS-Rapgef2, which are in turn activated by cAMP [52,53]. This raises the intriguing possibility that AC9 is a processing hub for cAMP signalling from other ACs. In another context, geranylgeraniol increased testosterone production in a testis tumour cell line. Its effect was eliminated by iRNA knock-down of AC9 [54]. Notably, Rap1 activity is fully dependent on geranylgeranylation, for which geranylgeraniol is the precursor [55].

#### 2.3.3. A-Kinase Anchoring Protein (AKAP)

A-kinase anchoring proteins are intracellular scaffolds serving the assembly of signalling domains [56]. In the case of AC9, AKAP79 (also known as AKAP5) and Yotiao (a product of the AKAP9 gene) have been shown to be in complex with AC9 [56]. The potential attachment of AC9 to AKAP79 is of considerable interest as, in addition to protein kinase A, this AKAP also binds calcineurin and has been shown to be involved in the phosphorylation/dephosphorylation cycles of integral membrane proteins [57,58]. The N-terminal domain of AC9 has been implicated in the interaction with AKAP79 [59]. Proximity biotinyl labelling from 2 to 3 fold overexpressed AC9 in mouse myocardiocytes also labelled AKAP79 [60]. With respect to Yotiao, the beta-adrenergic regulation of the IK potassium current is mediated by a complex of Yotiao and AC9 [61]. The structural details of the AC9–Yotiao interaction are not known. The same proximity labelling study of mouse neonatal cardiomyocytes did not report a labelling of Yotiao from AC9 even when beta adrenergic stimulation was applied [60]. However, it is of note that proximity labelling is an emerging technology that generates a large body of data requiring specialized statistical analysis.

#### 2.3.4. Popeye Domain Containing 1 (POPDC1)

Popeye domain containing 1 (POPDC1), also named Blood Vessel Epicardial Substance (BVES), is an evolutionary conserved membrane protein that binds cAMP. It is widely distributed in the body and has diverse functions in various tissues [62]. Two different groups have shown that POPDC1 can be co-immunoprecipitated with AC9 [63,64] in different cellular models. Li and co-workers also reported that when co-expressed in HEK293 cells, POPDC1 inhibited the basal activity of AC9 [63]. The other group found that AC9 and POPDC1 are involved in the regulation of the two-pore-domain potassium channel TREK1 in mouse hearts [64]. Neither report addressed the structural aspects of the AC9–POPDC interaction or the mode of action of POPDC1.

#### 2.3.5. Heat Shock Protein 20

The Dessauer laboratory reported that AC9 gene deletion in mice resulted in reduced phosphorylation of the small heat-shock protein Hsp20. They also provided evidence that AC9 directly binds to Hsp20 [15]. Interestingly, a proximity labelling study from the same investigators did not find Hsp20 as a protein in proximity to AC9 in neonatal mouse cardiomyocytes [60].

#### 2.3.6. Adenylyl Cyclases

A recent review on the oligomerization of transmembrane ACs highlights that transfected, i.e., overexpressed AC9 can form homodimers in COS-7 cells [31] as well as heterodimers with AC5 in neonatal cardiomyocytes [31]. The impact of such oligomerizations on enzyme function remains to be established. Interestingly, the C-terminal end of the C1b domain has been implicated as one of the contact surfaces supporting oligomerization [65]. This loop, located just before transmembrane helix 7 (TM7), contains two missense mutations, I772M [66] and S782G (Section 2.4), that reduce the basal activity of AC9.

### 2.4. Missense Mutations

**Figure 5:**
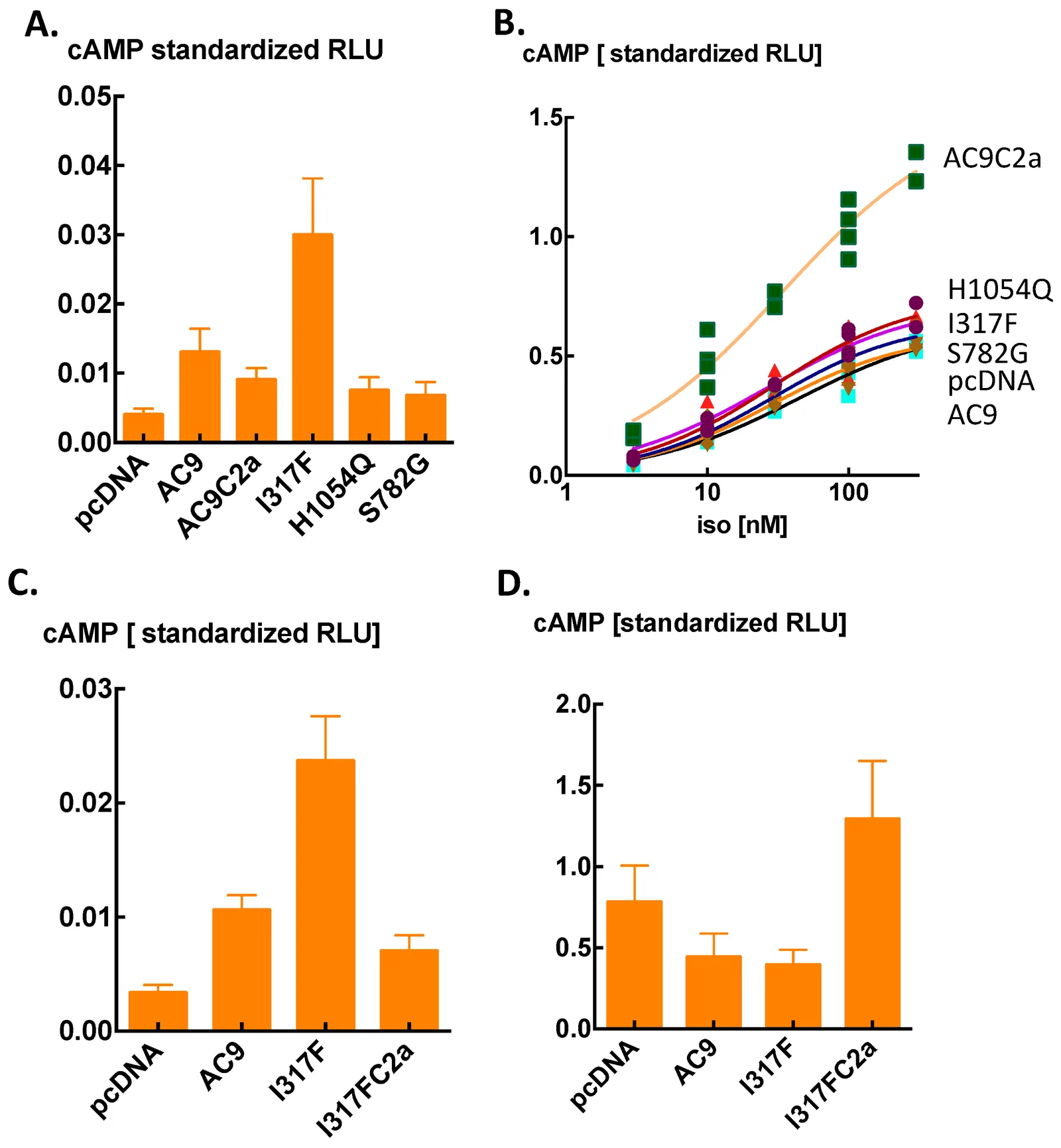
Effect of the missense human mutations listed in Table 1 on cAMP output by AC9 transiently expressed in MCF7 breast cancer cells. cAMP levels were detected by Glosensor22F luminescence [8]. Raw data were standardized by dividing with the RLU counts elicited by 5 µM forskolin/0.5 mM iso-butyl-methyl-xanthine-IBMX applied to each well. See ref. [8]. for further details. (**A**) Basal levels. The effects of all of the mutants tested were statistically significant *p* < 0.01. AC9C2a was run as validation sample: it is human AC9 with the C2b domain removed. One-way ANOVA of log-transformed values F (5, 78) = 128.5 *p* < 0.0001, followed by post hoc comparisons vs. AC9 with Dunnett’s test. Means ± S.D. n = 14/group (**B**) Response to isoproterenol (iso). Removal of the C2b domain enabled the AC9 response to iso as reported previously [8,9,21], whereas the other mutations were without effect. All datapoints are shown. (**C**,**D**) Further analysis of the stimulating effect of mutation I317F. Basal cAMP levels in (**C**) were all different from AC9, *p* < 0.001. One-way ANOVA of log-transformed values, F (3, 44) = 254.0 *p* < 0.0001, followed by post hoc comparisons vs. AC9 with Dunnett’s test. Means ± S.D. n = 12/group. (**D**) In contrast to the reduction in basal cAMP levels, removal of the C2b domain enhanced the effect of 10 nM iso on mutant I317F when compared with AC9 (*p* < 0.001) as well as the skeleton vector pcDNA (*p* < 0.05). One-way ANOVA of log-transformed values F (3, 16) = 19.75, *p* < 0.0001, followed by Tukey’s test for multiple comparisons. Means ± S.D., n = 4/group, unpublished results.

The assay of AC9 activity based on Glosensor22F is well suited for rapid screening of mutations. Figure 5 shows the results of an exemplar experiment. The mutations tested have been associated with but not causally linked to clinical phenotypes (Table 1). The results with I317F can be rationalized on the basis that it may change the conformation of the coiled-coil by forming a π-cation or hydrophobic interaction with Lys at position 321 (i, i + 4) [67] (Supplementary Figure S1A). The coiled coil starts at position 323. The effect was a two- to three-fold increase in basal cAMP levels (Figure 5A), but no change in the response to beta adrenergic stimulation (Figure 5B). Deletion of the C2b domain produced a drastic reduction in the basal activity below that of wild-type AC9 (Figure 5C) and markedly enhanced the response to isoproterenol (Figure 5D). As in the case of E326D (Figure 4), these results indicate that the positive allosteric efficacy of ARM governs basal cAMP production, while the autoinhibitory activity comes to the fore when Gsα is present.

The S782G mutation affects a juxtamembrane residue at the start of TM7 where the side chain of S782 establishes a hydrogen bond with T784 within the helix (Supplementary Figure S1B). Due to the absence of protons for hydrogen bonding and the greater flexibility of glycine, this mutation likely destabilizes the N-terminus of the helix of TM7. The response to isoproterenol did not change, but is mostly attributable to the host cell’s AC system. The result implies agreement with previous studies: that configuration of the transmembrane domains can modulate AC activity, most likely through changes in the conformation of the signalling helices arising from TM6 and TM12 [74,75].

Mutation H1054Q has a similar effect on cAMP production by AC9 as S782G. H1054 is at the start of the C2a catalytic domain. The His residue forms a hydrogen bond with L1120, which may maintain the position of a beta sheet which is part of the forskolin operated positive allosteric site (Supplementary Figure S1C). In the mutant, glutamine may not be suitable to fill in for the strict geometry of the His side-chain. This may reduce the efficacy of ARM at the allosteric site. As a result, basal activity driven by ARM is reduced. A similar loss of function due to the lack of a hydrogen bond upon a His to Asn mutation was reported for the major histocompatibility (MHC) class II molecule HLA-DR protein [76].

### 2.5. Pharmacons

As mentioned previously, in contrast to other AC isoforms, vertebrate AC9 is poorly stimulated by forskolin. The plausible reason is that the forskolin binding-pocket of AC9 has evolved to interact with the ARM in the C2b domain. Another feature of AC9 is the low sensitivity towards P-site inhibitors which are derivatives of adenosine [77,78,79].

Of further note is the stimulatory effect of oleate on AC9 [33], which is likely initiated by an interaction of oleate with the transmembrane arrays. A lipid binding-pocket has been discerned in the transmembrane arrays of human AC9 [10]. This is potentially the discovery of a novel class of receptor for lipids. Finally, the hydrophobic imidazole fungicide compound miconazole selectively stimulated AC9 in heterologous expression systems, and appeared to exert its effect through the transmembrane arrays [79,80].

### 2.6. Summary

Analysis of the activity changes of AC9 indicate a significant biological role of autoregulation. There are at least two structural elements of autoregulation, the coiled-coil formed between the signalling helices, and ARM, a short, highly conserved linear motif in the C2b domain. Autoregulation appears to have a dual role: autostimulation produces a cAMP signal, the destination(s) of which has(ve) not been established. In contrast, autoinhibition primarily gates the influence of extracellular stimuli on the activation of the enzyme. Autoinhibition is regulated—most likely in a cell-context dependent manner. In the heterologous expression systems where these features were observed, there is a 10- to 100-fold difference between the cAMP levels achieved by autostimulation and those evoked by Gs-coupled receptors. Whether or not this is also mirrored in various intracellular nanodomains for cAMP [81] remains to be elucidated. It is worth recalling here that in teleost fish, autoinhibition is the trait by which the *adcy9* ohnologs became subfunctionalized after whole-genome duplication, with the expression of the autoinhibited version encoded by *adcy9b* becoming largely restricted to the brain. Finally, Rap1 GTPase, which is activated by cAMP-responsive GTP exchange factors, can increase the activity of AC9—an exciting new finding.

## 3. Physiology and Pathology in Targeted Studies

### 3.1. Update After 2022

Work reporting the involvement of AC9 or *adcy9* in various physiologic and pathophysiologic processes has been reviewed previously [18,24,82]. Some selected interesting developments are as follows.

A potentially important study on the involvement of *adcy9* in regenerating planarian ganglia and, by extension, the regulation of neuronal survival has been already mentioned in the previous sections [20]. Two recent studies found *adcy9* and cAMP signalling to be upregulated in the context of new bone deposition of on artificial matrices [83,84]. No clear causal link between AC9 expression and bone formation was established. However, if there is such a link, it would be a significant incentive for producing specific stimulators of AC9 activity. A study of lung adenocarcinoma indicated that the *ADCY9* gene acts as a tumour suppressor to restrain the proliferation, migration, and invasion of the malignancy and can lead to a better survival or prognosis in patients [85]. Finally, a *tour de force* investigation showed that exosomes containing miRNA142-3p released by activated T cells suppress AC9 expression in the parenchymal cells of the salivary glands, thereby impairing secretory function and causing one of the key symptoms of Sjögren’s syndrome [86].

### 3.2. ADCY9 as a Pharmacogenetic Marker in Atherosclerosis

Dalcetrapib, an inhibitor of the cholesteryl ester transfer protein, is now in its third multi-million-dollar Phase III clinical trial. Post hoc analysis of the first trial indicated that an allele in intron 2 of the ADCY9 gene (rs1967309) determined the efficacy of the drug to reduce cardiovascular morbidity by affecting the atherosclerotic vascular intima thickening [87]. Subjects with the A/A genotype benefited from the drug, whereas those with G/G actually got worse; in heterozygotes, the drug had no discernible effect [87]. A company called DALCOR was set up to run a second Phase III trial of dalcetrapib using the genetic information gleaned from the first trial [88]. Other groups of investigators also ran genetically selective trials with evacetrapib [89] and anacetrapib [90], with negative results. However, the DALCOR trial claimed to have achieved positive outcomes that were statistically non-significant because of the COVID pandemic [88,91]. Although this conclusion has been openly challenged [92], a third dalcetrapib Phase III trial is currently underway.

The main critique of this endeavour is that the effect the rs1967309 alleles have on the expression of *ADCY9* mRNA or the protein product AC9 has never been published. The main objective clinical markers reported were the effect of serum on the efflux of cholesterol from J774 macrophages and the measurement of C-reactive protein in plasma [93]. Both of these parameters showed favourable changes in patients with A/A at rs1967309 and receiving dalcetrapib. However, there are no published data on how intron 2 regulates the expression or the activity of AC9. While, to their credit, DALCOR have conducted several studies of AC9 with the gene-trap mouse model bred to the C57/bl6 background [14,46], these studies simply cannot address the issue of the role of intron 2 in the human gene. Thus, while rs1967309 allele status may well be a useful predictor for the therapeutic efficacy of dalcetrapib, whether or not this has anything to do with some aspect of the workings of the *ADCY9* gene remains to be established.

## 4. Outlook to the Future

Research on AC9 would receive a major boost if specific modulators of its various activities were available or if the DALCOR trial was successful. On a different note, the pharmaceutical industry has produced a large number of inhibitors of cAMP hydrolysis. However, the source and regulation of the cAMP signal that is being amplified is rarely investigated. This is a pity, as combined targeting of cAMP compartments with modulators of cAMP synthesis as well as degradation could reduce the problems of unwanted side-effects. In the absence of specific pharmacons, localized gene knock-down technologies, e.g., viral delivery of antisense DNA, should be deployed to dissect the biological roles of AC9. A further major task is to discover which phosphorylations are functionally important and which protein kinases are involved. The chief puzzle of all is the significance of autoinhibition for brain function in vertebrates.

## Figures and Tables

**Table 1:** Missense mutations of human AC9 tested in Figure 5 (note that Gulsuner et al. [68] give the incorrect primary sequence position for CM138120).

HGMD Accession	HGMD Codon Change	Amino Acid Change	HGVS Nucleotide	HGVS Protein	Reported Phenotype	Reference
CM1931443	ATT-TTT	Ile317Phe	c.949A>T	p.I317F	Developmental disorder	[69,70,71]
CM138120	AGT-GGT	Ser782Gly	c.2344A>G	p.S782G	Schizophrenia	[68]
**GWAS catalogue**						
rs2230742		His1054Gln	c.3162T>G or c.3162T>A	p.H1054Q	Body height Depression	[72,73]

## Data Availability

All data are available on request from the author.

## Supplementary Materials

The following supporting information can be downloaded at https://www.mdpi.com/article/10.3390/cells15201836/s1, Figure S1: Possible structural basis of the effects of human missense mutations of AC9.
