# Supplementary material for: Adenylyl Cyclase 9: A Maverick Signalling Act in Search of Plots
Source: Cells. 2026 Oct 9;15(20):1836. doi: 10.3390/cells15201836 (PMC13703280; doi:10.3390/cells15201836)
Supplement: Supplementary file 1 [file cells-15-01836-s001.zip › cells-4589018-supplementary.pdf]

SUPPLEMENT

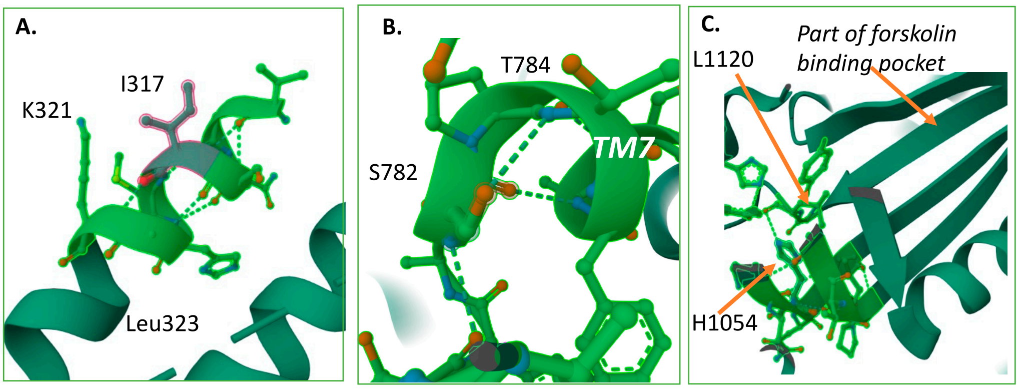

Supplementary Figure S1. Possible structural basis of the effects of human missense mutations of AC9. See main text for commentary. A. I317F, B. S782G, C. H1054Q Source: pdb_00009u3p (https://www.rcsb.org/3d-view/9U3P/1) was used to produce the images.
